# A Guide to Therapeutics

**Published:** 1883-07

**Authors:** 


*A Guide to Therapeutics.* By R. FARQUHARSON, M.P., M.D., F.R.C.P. Third Edition, pp. 373. London: Smith, Elder & Co., 1883.

The publication of three editions of this work within six years is sufficient testimony to the value which the profession puts upon it. The peculiarities which specially recommend it are its practical conciseness and the graphic manner in which the descriptive accounts of the actions of the various drugs are arranged. The Physiological and Therapeutical actions are arranged in parallel columns; and each column is subdivided into heads as the action of the drug on the various organs is described. The result is that the reason is appealed to and the memory assisted in a manner which must be peculiarly valuable to students.

